# Celebrity CEOs, digital transformation and firm performance in China: the moderating role of controlling shareholders and institutional investors

**DOI:** 10.3389/fpsyg.2023.1281553

**Published:** 2023-12-06

**Authors:** Rui Wang, Dong Shao, Xinliang Han, Yinyue Li

**Affiliations:** ^1^School of Business, Geely University of China, Chengdu, China; ^2^Business School, Northeast Normal University, Changchun, China

**Keywords:** celebrity CEO, digital transformation, firm performance, controlling shareholder, institutional investor

## Abstract

**Background:**

business leaders’ social status significantly impacts companies’ strategic direction and performance. Digital transformation, an important tool for companies to enhance competitiveness and resilience, plays an important role in the relationship between executive background and firm performance.

**Objective:**

To investigate the impact of celebrity chief executive officers (CEO) on firm performance through digital transformation.

**Method:**

Using data from companies listed on the main boards of the Shanghai and Shenzhen Stock Exchanges between 2017 and 2021, this study explored the relationship between celebrity CEOs, digital transformation, and firm performance.

**Result:**

Celebrity CEOs significantly enhanced a firm’s digital transformation. However, this effect weakened when controlling shareholders and institutional investors held more shares. Additionally, the study showed that celebrity CEOs can improve firm performance through digital transformation. These findings were robust across a range of sensitivity analyses.

**Conclusion:**

This study contributes to understanding celebrity CEOs’ decision-making motivations and economic impacts from a psychological perspective while also providing valuable insights for driving digital transformation within companies.

## 1 Introduction

A celebrity chief executive officer (CEO) is a manager who has achieved outstanding performance in leading a firm and has received representative social awards (Hayward et al., 2004; Wade et al., 2006). CEOs granted celebrity status attract social attention and elicit positive emotional reactions from stakeholders, making them representative figures of the organization and symbolic icons of the firm, thereby gaining social status and influence (Wade et al., 2006). Traditionally, celebrities are well-known actors or athletes in the film and sports industries (Rindova et al., 2006). With economic development, the public has shown strong interest in successful entrepreneurs. The rise of internet technology and modern media has provided the public with more convenient channels for obtaining information, further accelerating the celebrity status of business leaders (Treadway et al., 2009; Lovelace et al., 2018). As China has become the world’s second-largest economy, the Chinese government is increasingly emphasizing the role of entrepreneurs in economic development and believes that “The market vitality comes from people, especially from entrepreneurs, and from the entrepreneurial spirit. Chinese entrepreneurs have distinct characteristics of the times, national features, and world-class standards and have made significant contributions to promoting economic and social development and enhancing comprehensive national strength” (Central People's Government of the People's Republic of China, 2017). The status and influence of entrepreneurs in today’s society is growing. Previously, companies invited celebrities from the film or sports industry to endorse their products; however, CEOs often took the lead, increasing exposure to their companies through press conferences, interviews, social media, and other means (Bao et al., 2023). Examples include Steve Jobs (Apple), Bill Gates (Microsoft), Lei Jun (Xiaomi), and Dong Mingzhu (Gree).

The impact of celebrity CEOs on corporate strategy and economic performance has attracted extensive scholarly attention. Existing research suggests that celebrity CEOs can attract the attention of news media and stock analysts, convey signals to the public about a firm’s potential for good development, increase the organization’s attractiveness to exceptional job applicants, reduce premiums in mergers and acquisitions, promote greater corporate social responsibility, and boost stock prices(Fanelli and Grasselli, 2006; Cho et al., 2016; Shi et al., 2017; Wei et al., 2018; Bao et al., 2023). CEOs can also reap substantial personal benefits from celebrity status, including higher compensation levels, more stock options, seats on other firms’ boards, and better future job opportunities (Hayward et al., 2004; Wade et al., 2006). However, becoming a celebrity CEO is a double-edged sword. To maintain their social status and personal benefits, celebrity CEOs may engage in self-interest or even unethical behavior to continuously improve firm performance or attract social attention, which can have negative implications for the firm. These negative impacts include rent-seeking behavior, hiding negative information about the firm, and ultimately leading to a decline in firm performance (Malmendier and Tate, 2009; Cho et al., 2016; Saiyed et al., 2023). Other studies indicate that celebrity CEOs have only a short-term promotional effect on company performance and, in the long run, may cause negative market reactions (Wade et al., 2006). Numerous studies have examined whether celebrity CEOs have an inhibitory or promotional effect on firm performance. However, there is no consensus in the academic community. There is still a lack of in-depth research on the interests and motivations of celebrity CEOs in current popular firm strategies and whether they can translate high-risk and high-investment firm strategies into actual economic benefits. Digital transformation as a risk-oriented corporate strategy has not been explored in existing research on the impact of celebrity CEOs on promoting digital transformation or whether they can improve corporate performance through digital transformation.

In recent years, the digital transformation of industries has emerged as an effective pathway for firms to achieve innovation and sustainable development (Katsamakas, 2022). With continuous breakthroughs in digital technology and the rapid development of the digital economy, digital transformation can help companies undergo fundamental changes such as cost reduction, efficiency improvement, and enhanced innovation. This grants companies more robust economic vitality and significantly improve their performance levels (Li and Shen, 2021; Verhoef et al., 2021; Gupta and Bose, 2022), meeting celebrity CEOs’ high-performance level requirements. Digital economic development is a strategic choice and a key support for promoting high-quality economic development and building a new development paradigm in China (Xinhua News Agency, 2017). The 19th National Congress of the Communist Party of China explicitly stated the need to accelerate the construction of a digital China and build a new digitally-driven industrial ecosystem (Xinhua News Agency, 2017). The “14th Five-Year Plan and Long-Range Objectives through the Year 2035” emphasizes the need to accelerate digital development, promote digital industrialization and industrial digitalization, drive deep integration between the digital economy and the real economy, and create internationally competitive digital industry clusters (People's Republic of China, 2021). National and local governments have successively introduced a series of policy measures to promote the development of the digital economy, making the digital transformation of enterprises a focal point of attention across society (Zhang et al., 2022). As a micro-entity in a market economy, the digital transformation of enterprises is beneficial for driving innovation, enhancing internal control quality, optimizing corporate governance, and supporting the sustainable development of firms (Carroll et al., 2023). Implementing a digital transformation strategy not only allows companies to receive corresponding financial subsidies and tax reductions but also attracts the attention of stakeholders such as the public, capital markets, news media, and government departments, thus enhancing the reputation of celebrity CEOs (Zhang et al., 2022). Digital transformation is a typical risk-oriented strategy involving innovation and change. High uncertainty, high investment requirements, and long return cycles characterize it. Moreover, it is influenced by factors such as managers’ risk aversion, lagging management systems, business model inertia, and path dependence. As a result, digital transformation faces strong resistance in the business practice process, leading to a high failure rate of up to 90% for digital transformation in firms. This puts companies in a dilemma of “no transformation means death, transformation means seeking death” under the pressure of the digital economy (Oh et al., 2022). Existing research focuses on the strategic effectiveness and economic consequences of digital transformation, such as improving company efficiency, optimizing production processes, continuously innovating at the forefront of technology, maintaining market competitiveness, and influencing enterprises’ internationalization processes (Sousa and Rocha, 2019; Romero-Martínez and García-Muiña, 2021; Verhoef et al., 2021; Feliciano-Cestero et al., 2023). Some studies also examined digital transformation as a mediating variable to investigate the promoting effect of digital technology usage on firm performance (Tsou and Chen, 2023), and the promoting effect of Executive Overconfidence on Environmental Innovation (Zhou et al., 2022). Few scholars have explored the antecedents of corporate digital transformation. There is no consensus on the impact of digital transformation on firm performance (Li and Jia, 2018; Liu et al., 2023). Therefore, this study expands the relevant research on digital transformation from the level of effects to the level of antecedents, exploring the motivation of celebrity CEOs to engage in digital transformation and whether they can use digital transformation as a tool to improve performance.

In conclusion, to reflect the motivation of celebrity CEOs to engage in digital transformation, this study is based on social identity theory and upper echelons theory, revealing the economic consequences of the celebrity CEO from the perspective of digital transformation and corporate performance. It also explores the factors constraining celebrity CEOs and digital transformations. Using China’s A-share listed companies on the Shanghai and Shenzhen stock exchanges from 2017 to 2021 as the research sample, it empirically examines the relationship between celebrity CEOs, digital transformation, and firm performance by controlling for variables at the firm, CEO individual, and governance levels. The results show that celebrity CEOs can drive digital transformation in businesses and that the control of large shareholders and ownership by institutional investors constrains celebrity CEOs’ role in driving digital transformation. Furthermore, the fact that celebrity CEOs can effectively improve a company’s performance through digital transformation indicates that their motivation to drive digital transformation for the company primarily stems from the performance pressure associated with maintaining their celebrity status rather than seeking attention from the general public and stakeholders for personal gain. These findings are robust across a range of sensitivity analyses.

This study contributes to management theory in several ways. First, integrating social identity theory and upper echelons theory reveals the impact of celebrity CEO effects on digital transformation and firm performance. It interprets the mechanisms by which superstar managers influence organizational behaviors and outcomes from a psychological perspective. Second, this study reveals the factors driving digital transformation. It not only breaks through the theoretical understanding that executives inhibit digital transformation based on risk avoidance and path dependence but also proposes the viewpoint that celebrity CEO effects can counteract management’s psychological resistance. It expands the research on digital transformation from an outcome-oriented to a causal level. Finally, this study uncovers celebrity CEOs’ real purpose and underlying motivations in driving digital transformation in companies from a performance dimension. It verifies that the motivation for celebrity CEOs to promote digital transformation is not solely for their reputation but rather to effectively enhance the performance of the company. The study concludes that enhancing executives’ social influence is beneficial for companies to accelerate transformation, upgrading, and performance growth.

## 2 Literature reviews and hypotheses development

### 2.1 Influence of celebrity CEOs on firms’ digital transformation

The upper-echelon theory suggests that executive characteristics and life experiences shape cognitive patterns and value orientations, consequently influencing organizational behavior and strategic decision-making (Hambrick and Mason, 1984; Hambrick, 2007). Receiving representative social awards is a significant milestone in a CEO’s life, and attaining celebrity status is a key aspect of their social identity. As such, CEO’s motivations and preferences for decision-making can be influenced by their celebrity status, which is further manifested in their managerial and operational activities (Lee et al., 2020). While the upper-echelon theory does not delve into the underlying mechanisms by which executive characteristics impact corporate strategy and organizational behavior, social identity theory thoroughly interprets the roots of CEOs’ differentiated strategic decision-making. Social identity refers to individuals typically categorizing themselves within a specific social group and establishing guiding principles and standards of behavior based on the expectations bestowed upon that group (Tajfel, 1978). When individuals adhere to established standards within a group, they gain higher self-esteem and self-confidence, resulting in the motivation and action to maintain their status within the group (McCall, 1978; Burke, 1991; Stets and Burke, 2000). By acquiring societal awards, CEOs classify themselves within a group of business celebrities and then construct self-imposed standards that align with the expectations of the business celebrity’s identity, guiding their subsequent behaviors (Willer et al., 1989; Lovelace et al., 2018). As a result, celebrity CEOs approach corporate strategic decision-making with a more proactive attitude, aiming to meet the public’s perceived expectations and stakeholders (Wade et al., 2006; Zavyalova et al., 2017; Park et al., 2021). In addition to psychological motivating factors, celebrity status brings numerous additional benefits to the personal and corporate realms. For example, celebrity CEOs often enjoy higher compensation levels and expected future income (Graffin et al., 2008; Malmendier and Tate, 2009), and their personal authority and influence are significantly enhanced, enabling broader support for their preferred corporate strategies and the accumulation of various resources (Wade et al., 2006). The CEO’s celebrity effect confers advantages in maintaining supplier relationships, attracting top talent, and expanding financing channels (Hayward et al., 2004; Wade et al., 2006). The benefits and authority conferred by celebrity status generate strong motivation for CEOs, prompting them to take action to maintain or elevate their current social standing, which ultimately manifests in corporate strategies and organizational behavior (Hayward et al., 2004; Cho et al., 2016; Bao et al., 2023).

CEOs recognized as celebrities for their outstanding performance face higher internal aspirations and external expectations due to past achievements. However, if a firm’s performance declines or further improvement becomes challenging, the celebrity CEO’s identity and abilities as an exceptional manager may be questioned. The media’s excessive focus on celebrity CEOs amplifies the negative reactions of stakeholders, particularly the capital market, toward CEOs and firms. CEOs perceive these expectations and the severe consequences of not meeting them as leading to strong feelings of aversion to losses and a decline in performance (Wade et al., 2006; Mishina et al., 2010). With psychological aversion to losses and constant pressure to improve performance, celebrity CEOs face greater professional challenges. Since sustaining breakthroughs and achieving higher performance levels are difficult for already successful companies, bold actions are necessary to achieve these goals. Therefore, CEOs are motivated to implement risky strategies to protect their identities and status (Li and Zhang, 2022). Additionally, CEO celebrity status is attributed to society’s perception of a firm’s outstanding performance (Lovelace et al., 2018). The public and other stakeholders often attribute performance improvements to CEO’s exceptional management abilities (Chen et al., 2021). As celebrity CEOs internalize this attribution as self-recognition, they may develop excessive self-confidence and narcissistic tendencies (Hayward et al., 2004; Chatterjee and Hambrick, 2010). Excellent performance has led celebrity CEOs to believe in their invincible management abilities, considering that their knowledge and skills are superior to those of their peers. They also overestimate their control over corporate strategy and consciously choose challenging, high-risk decisions to match their identity as exceptional managers, further increasing the firm’s risk-taking behavior (Chatterjee and Hambrick, 2010; Hirshleifer et al., 2012; Li and Zhang, 2022).

Digital transformation refers to constructing a value-creation system driven by advanced digital technologies with data at its core. It can reshape strategic thinking, business processes, organizational structures, and business models to achieve close associations and value co-creation with stakeholders. This transformation aims to enhance the market competitiveness and performance levels of firms and is an inevitable choice for high-quality development in the era of the digital economy (Sousa and Rocha, 2019; Warner and Wäger, 2019). Digital transformation in firms effectively reduces overall costs, improves operational efficiency, enhances innovative capabilities, and comprehensively enhances operational performance and market value (Sousa and Rocha, 2019; Romero-Martínez and García-Muiña, 2021; Verhoef et al., 2021). It can fully meet the realistic needs of celebrity CEOs who constantly aim to improve their performance levels and maintain their celebrity identity and status. At the same time, digital transformation is a typical type of risk strategy characterized by high investment costs, long return cycles, high implementation difficulties, and significant uncertainties (Cortellazzo et al., 2019; Zhou et al., 2022). This aligns with the pursuit of risks and challenges driven by celebrity CEOs.

The development of the digital economy is a strategic choice and key support for promoting China’s high-quality economic development and constructing a new development pattern. National and local governments have successively introduced various policies and measures to promote the development of the digital economy, making digital transformation in firms the focus of attention in all sectors of society (Zhang et al., 2022). Firms undertaking digital transformation align with the current trend of socioeconomic development, are highly consistent with national policy guidelines, and release positive signals to internal and external organizations. This attracts the attention of the general public, government departments, and media. Companies undergoing digital transformation often have high expectations from capital markets, attracting many investors to follow suit. This enhances the market value of firms and generates a positive “exposure effect” for celebrity CEOs (Liu, 2015; Yang et al., 2023), further shaping the image of CEOs as visionary and outstanding managers. This satisfies celebrity CEOs’ need to maintain their identity and status through impression management (Lee et al., 2020). Therefore, celebrity CEOs may perceive digital transformation as a strategic action to maintain higher performance levels in the face of rising social expectations and performance pressure, as well as to sustain their celebrity identity and corresponding personal benefits (Liu et al., 2022). Celebrity CEOs may also be motivated by impression management and use the reputation effects of digital transformation to enhance their personal reputations, shaping themselves as visionary and bold managers among the public and stakeholders. Thus, Hypothesis 1 is proposed.

*H1*: Celebrity CEOs are positively associated with the degree of digital transformation in their firms.

### 2.2 The moderating role of controlling shareholders

Controlling shareholder refers to a shareholder in a limited liability company who owns more than 50% of the total capital of the company or holds more than 50% of the total share capital of a joint stock company or a shareholder whose contribution or shareholding is less than 50%, but whose voting rights based on their contribution or shareholding are sufficient to significantly influence the resolutions of shareholders’ meetings or shareholders’ general meetings (People's Republic of China, 2018). In China, listed companies generally exhibit concentrated ownership, with controlling shareholders holding high ownership stakes. The decision-making process of a firm’s management is inevitably influenced by controlling shareholders (Almazan et al., 2005; Zhang et al., 2021). Controlling shareholder influence has two main effects on the relationship between celebrity CEOs and digital transformation in companies. First, as the ownership stake of controlling shareholders increases, they have sufficient motivation to supervise and manage the management team to protect their shareholders’ interests (Wang et al., 2019; Iwasaki and Mizobata, 2020), thereby restraining opportunistic behavior and internal control issues within the management team and effectively alleviating the first type of agency problem (Jensen and Meckling, 1976). However, this also indirectly limits the CEO’s power in the process of business management (Lizares, 2022). The CEO’s initiatives and efforts also decrease with the strengthening of shareholder control (Shleifer and Vishny, 1986; Jiang and Kim, 2020), thereby weakening the role of celebrity CEOs in promoting digital transformation to preserve their identity and interests. However, in a dispersed ownership structure, no individual shareholder can exert absolute control over the firm, leading to free-riding behavior by numerous small shareholders (Grossman and Hart, 1980). The governance system of a firm faces difficulties in effectively supervising the management team, and the CEO is more likely to grasp the actual control of the firm, even build a corporate empire, and make strategic decisions that align with their own interests (Kothari et al., 2009), allowing celebrity CEOs to pursue digital transformation strategies that satisfy their personal celebrity identity needs without being constrained by shareholders.

Second, while controlling shareholders alleviates the first type of agency problem, they also give rise to the second type, which involves tunneling the firm and encroaching on the interests of minority shareholders (Shleifer and Vishny, 1997; Johnson et al., 2000). In China, the concentration of share ownership in companies is generally high, and effective mechanisms to restrain controlling shareholders have not yet been established in the capital market and listed companies, resulting in a higher probability of controlling shareholders engaging in activities that empty the firm compared with Western countries. This makes the agency’s problem of controlling shareholders encroaching on minority shareholders’ interests prominent (Saona et al., 2018; Jiang and Kim, 2020). An increase in the ownership stake of controlling shareholders leads to a “one-share dominance” phenomenon where controlling shareholders control the firm’s operational and management decisions, prioritizing their interests over those of the firm and other shareholders, thereby increasing the possibility of activities that empty the firm (Shleifer and Vishny, 1997; Johnson et al., 2000). Existing research shows that controlling shareholders not only engage in activities that empty the firm through related party transactions and the appropriation of firm funds but also reduce the level of internal control within the firm, thereby intensifying their pursuit of private interests in control (Fan and Wong, 2002). This encroaches on the limited resources available for strategic development and inhibits digital transformation strategies that rely heavily on firm resources. Therefore, Hypothesis 2 is proposed.

*H2*: The promotional effect of celebrity CEOs on the digital transformation of firms is weakened by controlling shareholders.

### 2.3 The moderating role of institutional investors

Institutional investors are legal entities that specializes in securities investment activities using their own funds or funds the general public raises (Fabozzi, 2011). With the rapid development of fund companies, securities companies, and other financial institutions, institutional investors have gradually become active in China’s capital market, playing a crucial role in aspects such as the equity structure, corporate governance, and strategic decision-making of listed companies (Tricker, 1998; He and Shen, 2014). Compared with other small shareholders and retail investors, institutional investors possess strong professional capabilities and industry backgrounds, significant information and resource advantages, and typically hold higher ownership stakes (Kahn and Winton, 1998). They not only intend to participate in overseeing management’s operational decisions but also can effectively intervene in the firm’s major strategies (Pound, 1988; Smith and Watts, 1992; Pu et al., 2023). Information asymmetry in the principal-agent relationship is the main source of monitoring costs for the firm, and institutional investors, often holding a higher proportion of shares, find the transaction costs associated with frequent buying or selling in the capital market are much higher than monitoring costs. Consequently, institutional investors tend to actively participate in corporate governance and management activities, thereby reducing potential agency costs by monitoring daily operations and strategic decision-making (Tricker, 1998; Borochin and Yang, 2017; Luo et al., 2023).

Institutional investors can participate in shareholder meetings as shareholders, elect board members and management, and participate directly in major personnel, finance, and strategy decisions (Fan et al., 2023). They can also exert external pressure on the firm’s management through capital market operations, exerting a “signaling” effect, effectively curbing the manager’s self-interest behavior, and causing management, represented by the CEO, to be more cautious in selecting the firm’s strategic activities (Aggarwal et al., 2011; Kacperczyk et al., 2015; Pu et al., 2023). Under the full supervision of institutional investors, celebrity CEOs have weakened their personal authority and discretionary power, making it difficult for them to engage fully in organizational activities that benefit their celebrity status and social standing. This also dampens the level of digital transformation promotion that they can undertake. Therefore, Hypothesis 3 is proposed.

*H3*: The promotion effect of celebrity CEOs on the digital transformation of firms is weakened by institutional investors.

### 2.4 The impact of celebrity CEOs on firm performance

The impact of celebrity CEOs on firm performance has not yet reached a consensus in academia and remains a research focus. Some scholars argue that CEO awards and celebrities improve short-term performance levels in companies. However, celebrity CEOs may reduce their involvement in management activities due to frequent social events and deal with a large number of non-core business matters, leading to a decline in subsequent firm performance that is detrimental to long-term development (Malmendier and Tate, 2009). Moreover, under excessive media hype, the discourse and control rights of celebrity CEOs within organizations greatly increase. They not only use their celebrity status to demand more monetized rewards but also make it difficult for the board of directors and shareholders to exercise limited supervision and checks, exacerbating the agency problem in the governance system and affecting firm performance (Yin et al., 2021). In addition, to continuously attract the attention of the public and media and consolidate their social status and vested interests, celebrity CEOs tend to promote many mergers and acquisitions to expand the firm’s size and influence. Under limited supervision and constraints, these mergers and acquisitions may fail to generate economic benefits for the firm (Shi et al., 2017). However, some scholars believe that social awards are scarce and exclusive and that awards conferred by authoritative institutions on CEOs can demonstrate their outstanding managerial abilities and good social reputation, thereby motivating and pressuring celebrity CEOs (Frey, 2007). While the public and media attribute a firm’s good performance to celebrity CEO, they also attribute any decline in performance to them (Chen et al., 2021). When a celebrity CEO fails to meet stakeholders’ expectations of continued performance growth, the firm’s board of directors often reduces the CEO’s generous compensation, and the CEO’s future compensation premium and career prospects in the external management market may be affected (Park et al., 2021). To maintain celebrity status and corresponding personal interests, celebrity CEOs must bear this attribution pressure and consistently maintain high levels of firm performance (Hayward et al., 2004; Wade et al., 2006; Graffin et al., 2013; Lee et al., 2020). Therefore, Hypothesis 4 is proposed.

*H4*: Celebrity CEOs are positively associated with firm performance.

### 2.5 The mediating role of digital transformation between celebrity CEOs and firm performance

Digital transformation, as a profound strategic change for organizations, often faces numerous obstacles in the implementation process and requires top-down support to drive the transformation forward (Zhang et al., 2022). Managers’ understanding and support of digital transformation not only satisfy the high demand for resources in the transformation process but also bring about changes in attitudes, capabilities, behaviors, and performance at the individual, group, and organizational levels, enhancing the firm’s ability to absorb and utilize digital technologies (Cichosz et al., 2020; Firk et al., 2022). CEOs who advocate digital transformation tend to actively embrace rapid and disruptive changes in the digital economy and gain insight into digital technology applications’ transformative and value-enhancing capabilities in businesses. They also develop appropriate strategies for digital transformation, recruit or cultivate digital talent, build dedicated digital teams, and make long-term strategic commitments and financial investments, thereby controlling the conversion costs associated with introducing digital technologies and reducing path dependence and resistance to managerial inertia. This effectively mitigates conflicts of interest in the digital transformation process (Verhoef et al., 2021). A company’s performance and value can be rapidly enhanced through digital transformation in several ways. First, it can reduce costs and improve efficiency by reducing various costs, such as production and management costs, while also enhancing production, operation, and management efficiency (Romero-Martínez and García-Muiña, 2021; Feliciano-Cestero et al., 2023). Second, it can strengthen the company’s capabilities, including the overall innovation and absorptive capacity and the individual cognitive, learning, and adaptability capabilities of internal employees (Sousa and Rocha, 2019; Gupta and Bose, 2022). Third, it can drive business innovation, mainly technological, managerial, and product innovation, among other aspects (Sousa and Rocha, 2019; Li and Shen, 2021).

Therefore, Hypothesis 5 is proposed.

*H5*: Celebrity CEOs enhance firm performance by promoting digital transformation.

**Figure 1:**
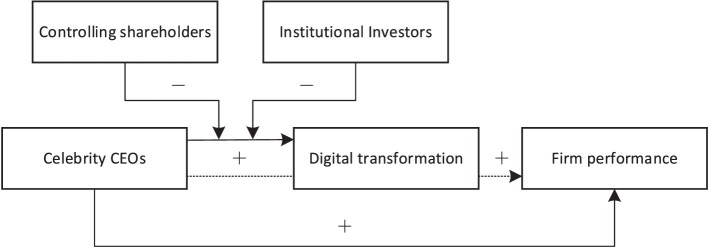
Conceptual framework. *Note: The dashed line represents the mediating effect.

The conceptual framework of this study is illustrated in Figure 1.

## 3 Materials and methods

### 3.1 Sample selection and data sources

This study focused on companies listed on the main board of A-shares in China’s Shanghai and Shenzhen stock markets from 2017 to 2021. The main board companies were selected because they are more mature and stable than other market segments. The disclosure of the data and information was clear, transparent, and reliable. Additionally, the main board-listed companies tend to have a larger scale and social influence, making it easier for CEOs to obtain various representative social awards. Data on celebrity CEOs were manually collected and compiled from personal resources, interviews, firm annual reports, firm websites, social media platforms, news reports, and Internet search engines. Other firm and CEO data were obtained from the CSMAR database and the Shanghai and Shenzhen stock exchange websites. After obtaining relevant data for the sample, the following steps were taken. First, financial and insurance companies were excluded due to the specific and complex nature of their financial indicators and business objectives. Second, exclude companies with “ST” and “*ST.” When a Chinese A-share listed company incurs consecutive losses for more than 2 years or faces other significant problems, the stock exchange will subject the company to special treatment by adding a “ST” and “*ST” designation. These companies often exhibit abnormal financial performance, increased risk, and potential governance issues, which may skew the analysis results. Third, CEO samples with a tenure of less than 12 months were excluded because shorter tenures are less likely to significantly impact firm strategy. Fourth, firms with incomplete key data were excluded. Finally, all continuous variables were wonsorized at 1 and 99% levels to eliminate the potential influence of outliers. This study obtained a sample of 13,126 “firm-year” observations.

### 3.2 Measures

#### 3.2.1 Dependent variable

Tobin’s Q, an important indicator of firm market value, is widely used in studies on firm performance.

#### 3.2.2 Independent variable

The independent variable in this study is celebrity CEOs (CCEO). The measurement method for celebrity CEOs in the existing literature originated from the development of scholars on whether CEOs have received top media business awards (Malmendier and Tate, 2009; Cho et al., 2016). Studies in China have adopted the annual business awards issued by the top five social media platforms to define celebrity CEOs (Lovelace et al., 2018). However, this measurement method overlooks the importance and representativeness of awarding bodies in China’s specific institutional and economic context. Awards granted by various government departments in China often have more influence and authority than the media. Additionally, existing measurement methods include only a few top influential business awards, which limits the definition of celebrity CEOs to a small number of well-known executives. However, CEOs receiving various representative awards at different levels are granted different degrees of celebrity status; even if this status is limited to the firm’s local area or industry, it can still impact the CEO’s strategic decision-making and firm behavior. Therefore, to improve the broad representativeness of the research subjects and the applicability of the Chinese context, this study extends the existing measurement method of celebrity CEOs by using various representative awards granted by the government and mainstream media to CEOs as the standard for defining celebrity CEOs. If a CEO has received representative social awards issued by the national, provincial, municipal, or mainstream media before year t, including Outstanding Entrepreneur, Outstanding Youth, Model Worker, May 1st Labor Medal, and March 8th Red Flag Bearer, the CEO is assigned a value of 1; otherwise, 0.

#### 3.2.3 Mediating variable

Previous research has shown that the content and frequency of occurrence in the annual reports of listed companies reflect their business philosophy, development plans, and strategic directions. Digital transformation, an important strategy for high-quality development in the current digital economy, is reflected in annual reports as an indication of a firm’s willingness, motivation, and progress toward digital transformation. This study refers to the measurement method of corporate digital transformation in related research, which quantifies the degree of digital transformation for listed companies by calculating the frequency of “digital transformation” related content in annual reports. Using Python data scraping techniques and text feature word analysis methods, five key words that reflect digital transformation in annual reports are extracted: “artificial intelligence technology,” “blockchain technology,” “cloud computing technology,” “big data technology,” and “digital technology application.” After removing prefixes or negations unrelated to the firm, the frequency of occurrence of these keywords in the annual report was calculated, and the natural logarithm was taken after adding one to obtain the digital transformation level (DT) for each sample firm (Gupta and Bose, 2022).

#### 3.2.4 Moderating variables

The moderating variables are Top Shareholder Control (Top) and institutional investor shareholdings (Ins). Specifically, top shareholder control is measured as the percentage of shares held by the largest shareholder at the end of the year, calculated as the ratio of the number of shares held by the largest shareholder to the number of shares in circulation (Gul et al., 2010). Institutional investor shareholding is the percentage of shares held by institutional investors at the end of the year, calculated as the ratio of the number of shares held by institutional investors to the number of shares in circulation (An and Zhang, 2013; Callen & Fang, 2013).

#### 3.2.5 Control variables

Referring to relevant existing research (Pathan, 2009; Feng et al., 2011), this study controlled other factors that may influence the regression analysis, including firm size, firm age, cash flow ratio, total asset turnover ratio, leverage, financial slack, human resource slack, state-owned enterprises, CEO gender, CEO age, dual roles, executive pay gap, executive political connections, board size, board independence, industry, and year. Table 1 presents the definitions of the main variables.

**Table 1:** Definition and measurement of variables.

Variable name	Symbol	Measurement method
Firm performance	Tobin Q	Market value to net assets
Celebrity CEOs	CCEO	CEO received awards = 1, and 0 otherwise
Digital transformation	DT	Ln (the frequency of occurrence of keywords +1)
Top shareholder control	Top	The ratio of the number of shares held by the largest shareholder
Institutional investor shareholding	INS	The ratio of the number of shares held by institutional investors
Firm size	FS	Ln (the firm’s total assets)
Firm age	FA	Current year - year of establishment of the firm +1
Cash flow ratio	Cash	The ratio of net cash flow from operating activities to total assets
Total asset turnover ratio	TAT	The ratio of net sales revenue to total assets of the firm
Leverage	Lev	The ratio of total liabilities to total assets
Financial Slack	FSlack	Current cash and cash equivalents to total assets adjusted for industry average
Human Resources Slack	HRSlack	Current employee count to total sales adjusted for industry average
State-owned enterprises	SOE	State-owned enterprises = 1, and 0 otherwise
CEO gender	Male	the CEO was male = 1, and 0 otherwise
CEO age	Age	the actual age of the CEO
Dual role	Dua	The general manager was also the chairman of the board = 1, and 0 otherwise
Executive pay gap	Gap	The ratio of the total compensation of the top three executives to the total compensation of all executives
Political connection	PC	The CEO had political connection = 1, and 0 otherwise
Board size	BS	Total number of board members
Board independence	BI	The ratio of independent directors on the board
Industry	Industry	Industry dummy variable
Year	Year	Year dummy variable

### 3.3 Model design

This study employed a fixed-effects model that controls for industry and year effects to conduct hypothesis testing. Equation (1) examines the impact of celebrity CEOs on the degree of digital transformation of firms. Equations (2) and (3) extend Equation (1) by including moderating variables, explanatory variables, and the interaction term between the explanatory and moderating variables to construct a moderated-effects model. This model investigates whether the effect of Celebrity CEOs on digital transformation is influenced by top shareholder control and institutional investor shareholdings. Equation (4) examines celebrity CEOs’ impact on firm performance. Equations (4) and (5) use a stepwise method to construct a mediation model to examine Celebrity CEOs’ influence on firm performance and the mediating role of digital transformation. Existing research suggests that changes in firm performance lag strategic decision-making and implementation; therefore, a one-period lag is applied to the dependent variable of firm performance.


(1) $$DT_{i.t}=\alpha_{0}+\alpha_{1}CCEO_{i.t}+\alpha Controls_{i.t}+\varepsilon$$

(2) $$\begin{array}{l} DT_{i.t}=\alpha_{0}+\alpha_{1}CCEO_{i.t}+\alpha_{2}Top_{i.t}\\ \;\;\;\;\;\;\;\;\;\;\;\;\;\;\;+\alpha_{3}CCEO_{i.t}*Top_{i.t}+\alpha Controls_{i.t}+\varepsilon \; \end{array}$$

(3) $$\begin{array}{l} DT_{i.t}=\alpha_{0}+\alpha_{1}CCEO_{i.t}+\alpha_{2}INS_{i.t}\\ \;\;\;\;\;\;\;\;\;\;\;\;\;\;\;+\alpha_{3}CCEO_{i.t}*INS_{i.t}+\alpha Controls_{i.t}+\varepsilon \end{array}$$

(4) $$TobinQ_{i.t+1}=\alpha_{0}+\alpha_{1}CCEO_{i.t}+\alpha Controls_{i.t}+\varepsilon$$

(5) $$TobinQ_{i.t+1}=\alpha_{0}+\alpha_{1}CCEO_{i.t}+\alpha_{2}DT_{i.t}+\alpha Controls_{i.t}+\varepsilon$$

In the equations, *i* and *t* represent firms and years, respectively, *α_0_* represents intercept, *α* represents regression coefficients of each explanatory variable. *ε* denotes the residual value.

## 4 Results

### 4.1 Descriptive statistics and correlation analysis

Table 2 presents descriptive statistics for the main variables in this study, including number of observations, means, medians, standard deviations, minimum values, and maximum values. The average Tobin’s Q, which reflects the performance level of the sample firms, was 2.234. The mean value of the degree of digital transformation was 1.132. Only 5% of the sample firms have CEOs with celebrity status, indicating that opportunities for CEOs to receive various social awards are still limited. The mean value of the shareholding percentage of the largest shareholder was 34.6%, and the mean value of institutional investors’ shareholding was 37.4%.

**Table 2:** Descriptive statistical analysis.

Variable	Observations	Mean	S.D.	Min	Median	Max
Tobin Q	13,126	2.234	1.816	0.815	1.546	17.68
DT	13,126	1.132	0.693	0.000	1.266	4.595
CCEO	13,126	0.050	0.000	0.000	0.217	1.000
Top	13,126	0.346	0.327	0.084	0.147	0.755
INS	13,126	0.374	0.379	0.000	0.238	0.882
FS	13,126	22.100	21.930	19.520	1.272	26.110
FA	13,126	2.829	2.890	1.792	0.324	3.497
Cash	13,126	0.041	0.041	−0.196	0.070	0.257
TAT	13,126	0.637	0.539	0.053	0.432	2.777
Lev	13,126	0.418	0.403	0.0460	0.210	0.925
FSlack	13,126	0.000	−0.0310	−0.164	0.118	0.405
HRSlack	13,126	−0.001	−0.004	−0.017	0.01	0.039
SOE	13,126	0.333	0.000	0.000	0.471	1.000
Male	13,126	0.939	1.000	0.000	0.240	1.000
Age	13,126	49.600	50.000	27.000	6.438	80.000
Dua	13,126	0.281	0.000	0.000	0.449	1.000
Gap	13,126	0.411	0.392	0.000	0.125	1.000
PC	13,126	0.848	1.000	0.000	0.359	1.000
BS	13,126	2.128	2.197	1.609	0.197	2.708
BI	13,126	0.375	0.333	0.333	0.0530	0.600

Table 3 presents the results of the correlation tests. The correlation coefficient between the variables was <0.600. The correlation coefficient between celebrity CEOs and digital transformation was 0.087 and significant at the 5% level, indicating a positive correlation between celebrity CEOs and digital transformation. The correlation coefficient between digital transformation and firm performance was 0.041 and significant at the 5% level, suggesting a positive correlation between digital transformation and firm performance. We also ran the variance inflation factor (VIF) test, and all variables had VIF values well below the standard value of 10, indicating that multicollinearity did not bias the research results.

**Table 3:** Correlation analysis.

No.	Variable	1	2	3	4	5	6	7	8	9	10	11	12	13	14	15	16	17	18	19	20
1	TobinQ	1.000																			
2	DT	0.041*	1.000																		
3	CCEO	−0.012	0.087*	1.000																	
4	Top	−0.088*	−0.096*	−0.018*	1.000																
5	INS	−0.034*	−0.040*	0.032*	0.342*	1.000															
6	FS	−0.441*	0.017	0.081*	0.207*	0.462*	1.000														
7	FA	−0.061*	−0.051*	−0.029*	−0.078*	0.138*	0.148*	1.000													
8	Cash	0.050*	−0.021*	0.007	0.101*	0.083*	0.025*	−0.029*	1.000												
9	TAT	−0.049*	0.076*	0.011	0.089*	0.079*	0.046*	0.004	0.130*	1.000											
10	Lev	−0.248*	−0.081*	0.022*	0.073*	0.264*	0.536*	0.198*	−0.192*	0.120*	1.000										
11	FSlack	0.143*	0.057*	−0.019*	0.042*	−0.057*	−0.195*	−0.037*	0.184*	−0.006	−0.347*	1.000									
12	HRSlack	0.146*	0.034*	−0.008	−0.079*	−0.121*	−0.288*	−0.003	−0.045*	−0.275*	−0.158*	0.047*	1.000								
13	SOE	−0.141*	−0.149*	0.011	0.223*	0.402*	0.374*	0.233*	−0.000	0.049*	0.313*	−0.047*	−0.092*	1.000							
14	Male	−0.024*	−0.005	0.000	−0.013	0.025*	0.040*	−0.022*	−0.016	0.012	0.029*	−0.013	−0.018*	0.052*	1.000						
15	Age	−0.030*	−0.068*	0.051*	0.046*	0.084*	0.103*	0.100*	0.043*	−0.002	0.025*	−0.009	0.004	0.109*	0.028*	1.000					
16	Dua	0.052*	0.085*	0.056*	−0.035*	−0.204*	−0.178*	−0.117*	−0.003	−0.018*	−0.137*	0.052*	0.050*	−0.285*	0.025*	0.128*	1.000				
17	Gap	0.160*	0.002	−0.020*	0.011	−0.041*	−0.221*	0.059*	0.005	0.002	−0.107*	0.103*	0.030*	−0.125*	−0.053*	0.015	0.181*	1.000			
18	PC	−0.040*	−0.032*	0.026*	0.026*	0.070*	0.094*	−0.003	0.002	0.009	0.062*	−0.027*	−0.020*	0.077*	−0.003	0.034*	−0.048*	−0.057*	1.000		
19	BS	−0.140*	−0.055*	0.002	0.015	0.192*	0.271*	0.087*	0.035*	0.025*	0.168*	−0.056*	−0.052*	0.255*	0.072*	0.056*	−0.177*	−0.242*	0.121*	1.000	
20	BI	0.045*	0.055*	0.031*	0.041*	−0.045*	−0.010	−0.049*	−0.013	−0.034*	−0.020*	0.030*	0.017	−0.066*	−0.063*	0.014	0.113*	0.090*	−0.022*	−0.546*	1.000

*indicates *p* < 0.05.

### 4.2 Hypothesis testing

Table 4 presents the regression results for celebrity CEOs, digital transformation, and firm performance. In Column (1), the regression coefficient of celebrity CEOs on the degree of digital transformation was significantly positive (*β* = 0.300, *p* < 0.01), indicating that celebrity CEOs have a promotional effect on the digital transformation of firms, thus supporting H1. In Column (2), the interaction term between celebrity CEOs and the shareholding percentage of the largest shareholder was significantly negative (*β* = −0.886, *p* < 0.01), suggesting that the shareholding percentage of the largest shareholder plays a negative moderating role in the relationship between celebrity CEOs and digital transformation. Specifically, when the largest shareholder’s control level is high, celebrity CEOs’ promotional effect on digital transformation is suppressed, thus verifying H2. In Column (3), the interaction term between celebrity CEOs and the shareholding percentage of institutional investors was significantly negative (*β* = −0.372, *p* < 0.1), indicating that institutional investors’ shareholding plays a negative moderating role in the relationship between celebrity CEOs and digital transformation. Specifically, when institutional investor ownership is high, celebrity CEOs’ impact on digital transformation is inhibited, thus validating H3.

**Table 4:** Regression results of celebrity CEOs, digital transformation and firm performance.

Variable	(1)	(2)	(3)	(4)	(5)
	DT_t_	DT_t_	DT_t_	TobinQ_t + 1_	TobinQ_t + 1_
CCEO_t_	0.300***	0.589***	0.449***	0.105**	0.082**
	(6.232)	(5.324)	(4.453)	(2.575)	(1.983)
Top_t_		−0.333***			
		(−5.124)			
CCEO_t_*Top_t_		−0.886***			
		(−3.126)			
INS_t_			0.188***		
			(4.135)		
CCEO_t_*INS_t_			−0.372*		
			(−1.808)		
DT_t_					0.078***
					(7.177)
_cons	−2.391***	−2.370***	−2.156***	11.008***	11.195***
	(−8.668)	(−8.607)	(−7.580)	(28.079)	(28.454)
Controls	Control	Control	Control	Control	Control
Industry/Year	Control	Control	Control	Control	Control
N	13,126	13,126	13,126	13,126	13,126
Adj R^2^	0.345	0.347	0.346	0.333	0.336

(1) *indicates *p* < 0.1, **indicates *p* < 0.05, ***indicates *p* < 0.01; (2) Figures in parentheses are standard error values.

Further analysis was conducted to determine whether celebrity CEOs can improve firm performance through digital transformation. This study analyzed the economic consequences of celebrity CEOs leading to digital transformation, including their impact on firm performance and the mediating role of digital transformation. Based on the mechanism of the mediating effect, a stepwise method was employed to test the regression coefficients in Columns (4) and (5) to identify the mediating role of digital transformation. In Column (4), the regression coefficient of celebrity CEOs on firm performance was significantly positive (*β* = 0.105, *p* < 0.05), indicating that celebrity CEOs can enhance firm performance, which supported H4. In Column (5), both the regression coefficient of celebrity CEOs on firm performance (*β* = 0.082, *p* < 0.05) and the regression coefficient of digital transformation on firm performance (*β* = 0.078, *p* < 0.01) were significantly positive, suggesting that there is a mediating effect of digital transformation in the transmission path from celebrity CEOs to firm performance. Therefore, H5 was supported. A Sobel test was conducted to assess the mediation effect of digital transformation. The Z-values of the test statistics were significant at the 1% level, consistent with the conclusions drawn from the stepwise mediation analysis.

### 4.3 Robustness test

#### 4.3.1 Endogeneity test

Previous research suggests that the CEO receiving prestigious social awards may not only be a unilateral action by the awarding body but may also be influenced by factors that the firm or CEO actively seeks. This leads to the self-selection of CEOs. Propensity score matching (PSM) method was employed to overcome endogeneity problem for robustness testing. After matching the samples using a 1:4 nearest neighbor matching method, regression analysis was conducted again on the matched samples using Models (1)–(5). Owing to space limitations, only the results of the second stage are reported here. The results of the PSM robustness tests are listed in Table 5. The results showed that celebrity CEOs have a promotional effect on firms’ digital transformation, whereas controlling shareholders and institutional investor shareholdings have a negative moderating effect. Digital transformation continues to play a mediating role in the relationship between celebrity CEOs and improved firm performance. Therefore, the research findings are robust and reliable.

**Table 5:** PSM robustness test.

Variable	(1)	(2)	(3)	(4)	(5)
	DT_t_	DT_t_	DT_t_	TobinQ_t + 1_	TobinQ_t + 1_
CCEO_t_	0.315***	0.616***	0.506***	0.077*	0.055
	(6.002)	(5.026)	(4.686)	(1.682)	(1.204)
Top_t_		−0.196			
		(−1.298)			
CCEO_t_*Top_t_		−0.909***			
		(−2.947)			
INS_t_			0.294***		
			(2.771)		
CCEO_t_*INS_t_			−0.475**		
			(−2.173)		
DT_t_					0.067***
					(3.692)
_cons	−1.747***	−1.690***	−1.497***	8.905***	9.023***
	(−3.219)	(−3.124)	(−2.682)	(16.619)	(16.838)
Controls	Control	Control	Control	Control	Control
Industry/Year	Control	Control	Control	Control	Control
N	2,882	2,882	2,882	2,882	2,882
Adj R^2^	0.369	0.372	0.371	0.350	0.353

(1) *indicates *p* < 0.1, **indicates *p* < 0.05, ***indicates *p* < 0.01; (2) Figures in parentheses are standard error values.

#### 4.3.2 Sample selection Bias test

The appointment of celebrity CEOs is a voluntary subjective action, and not all companies are inclined to appoint such CEOs. This led to a sample selection bias. The Heckman two-stage method was employed to overcome this problem. Previous research suggests that advertising expenses (ADR) can significantly suppress the frequency of negative vocabulary used by the media in describing companies, allowing firms to gain media attention and favor, thereby increasing the likelihood of CEOs receiving various prestigious social awards. Advertising expenses are the ratio of year-end sales expenses to the operating income. In the first stage, ADR was used as an instrumental variable for celebrity CEOs to explore the factors influencing the selection of celebrity CEOs by listed companies. The Probit model was used to calculate the probability of listed companies appointing celebrity CEOs, i.e., the inverse Mills ratio (IMR). The IMR was then included as an explanatory variable in the second-stage regression Models (2)–(5) for digital transformation and firm performance dependent variables. The test results are listed in Table 6. In Column (1), the regression coefficient of the instrumental variable ADR on celebrity CEOs was significantly positive (*β* = 1.472, *p* < 0.01), and the IMR coefficient was significant in Columns (2)–(6), indicating the presence of sample selection bias in the model. The Heckman two-stage regression method is robust and effective. The results in Columns (2)–(6) showed that celebrity CEOs can promote a firm’s digital transformation while controlling shareholder and institutional investors’ shareholdings inhibited the promotional effect of celebrity CEOs on digital transformation. Digital transformation continues to play a mediating role in the relationship between celebrity CEOs and improved firm performance. Therefore, the research findings are robust and reliable.

**Table 6:** Heckman two-stage robustness test.

Variable	(1)	(2)	(3)	(4)	(5)	(6)
	CCEO_t_	DT_t_	DT_t_	DT_t_	TobinQ_t + 1_	TobinQ_t + 1_
ADR_t_	1.472***					
	(6.628)					
CCEO_t_		0.280***	0.584***	0.449***	0.088**	0.067
		(5.816)	(5.335)	(4.456)	(2.161)	(1.640)
Top_t_			−0.351***			
			(−5.112)			
CCEO_t_*Top_t_			−0.934***			
			(−3.339)			
INS_t_				0.192***		
				(4.073)		
CCEO_t_*INS_t_				−0.421**		
				(−2.056)		
DT_t_						0.074***
						(6.471)
IMR		−1.099***	−1.111***	−1.090***	−0.788***	−0.707***
		(−11.416)	(−11.524)	(−11.271)	(−5.508)	(−4.853)
_cons	−7.315***	5.300***	5.405***	5.469***	16.412***	16.022***
	(−12.731)	(7.244)	(7.378)	(7.463)	(15.599)	(15.072)
Controls	Control	Control	Control	Control	Control	Control
Industry/Year	Control	Control	Control	Control	Control	Control
N	12,350	12,350	12,350	12,350	12,350	12,350
Adj R^2^	0.088	0.352	0.355	0.353	0.327	0.330

(1) *indicates *p* < 0.1, **indicates *p* < 0.05, ***indicates *p* < 0.01; (2) Figures in parentheses are standard error values.

#### 4.3.3 Lagged variable test

Considering the potential lagging effects of celebrity CEOs’ decision preferences and strategic choices on digital transformation and firm performance, this study conducted robustness tests by lagging digital transformation by one period and lagging independent and control variables by two periods and then reintegrating them into Models (1)–(5) for analysis. The test results showed that celebrity CEOs still have a promotional effect on firms’ digital transformation, while the negative moderating effect of controlling shareholders and institutional investors’ shareholdings remains unchanged. The degree of digital transformation continued to play a mediating role in the relationship between celebrity CEOs and improved firm performance. Thus, the research findings are robust and reliable. Owing to space limitations, the regression results are omitted.

#### 4.3.4 Sample range replacement test

This study employed two methods to replace the sample range of the research objects. First, it narrowed the sample range to manufacturing companies and conducted regression analysis using Models (1)–(5). The test results are consistent with this hypothesis. Second, it narrowed the sample time interval to 2019–2021 and reintegrated it into Models (1)–(5) for analysis. The robustness test results for replacing the sample range are consistent with this hypothesis. There were no significant differences between the results of the regression analysis with the replacement sample range and those presented earlier, indicating that the findings of this study remain robust. Owing to space limitations, the regression results are omitted.

## 5 Discussion

The impact of celebrity CEOs on business strategy and economic performance has garnered widespread scholarly attention. Although existing research has made preliminary explorations from various dimensions such as corporate social responsibility, mergers and acquisitions, risk-taking, and negative information concealment (Cho et al., 2016; Shi et al., 2017; Chen et al., 2021; Liu et al., 2022), it does not align with the current pace of commercialization of business leaders. There is a lack of in-depth research on celebrity CEOs’ interests and motivations for current popular business strategies and whether they can translate high-risk, high-investment strategies, such as digital transformation, into actual economic benefits. Moreover, there is no consensus in the academic community on whether celebrity CEOs have an inhibitory or promotional effect on firm performance. Some scholars argue that celebrity CEOs can enhance company performance and bring about excess returns (Nguyen, 2015; Bao et al., 2023), while others suggest that celebrity CEOs may engage in rent-seeking behavior or conceal negative information about the company, leading to a decline in firm performance (Malmendier and Tate, 2009; Cho et al., 2016; Saiyed et al., 2023).

Therefore, based on previous research, this study on digital transformation as a strategic vehicle to address these issues examined the economic consequences of celebrity CEOs driving digital transformation. Consistent with our theoretical perspective, we found that, based on the upper-echelons and social identity theories, celebrity CEOs under performance pressure to maintain their celebrity status are likely to drive risky strategies to enhance company performance (Wade et al., 2006; Zavyalova et al., 2017; Park et al., 2021). Digital transformation is characterized by high input costs, long return cycles, implementation difficulties, and high uncertainty but significant returns, which align with the risk-seeking behavior of celebrity CEOs (Li and Shen, 2021; Verhoef et al., 2021; Gupta and Bose, 2022). Therefore, celebrity CEOs may see digital transformation as a strategic action to maintain high firm performance levels under increasing societal expectations and pressure to maintain their celebrity status and the corresponding personal benefits. However, controlling shareholders may indirectly limit the power of CEOs in the management process to curb opportunistic behavior and internal control issues (Shleifer and Vishny, 1986; Jiang and Kim, 2020; Lizares, 2022), and the asset-stripping behavior of major shareholders may erode the limited resources available for strategic development, thereby suppressing the digital transformation strategy that relies heavily on corporate resources (Fan and Wong, 2002). Simultaneously, under the full supervision of institutional investors, celebrity CEOs’ authority and discretion may weaken (Aggarwal et al., 2011; Kacperczyk et al., 2015; Pu et al., 2023), which may also inhibit the degree to which digital transformation is promoted.

### 5.1 Theoretical contributions

This study contributes to management theory in several ways. First, integrating social identity theory and upper-echelons theory reveals the impact of CEOs’ celebrity effects on digital transformation and firm performance. It interprets the mechanisms by which managers influence organizational behavior and outcomes from a psychological perspective, thus opening the black box of how executive characteristics influence corporate strategies. In addition, it expands the micro-level research boundaries of executive personal characteristics from a social dimension, providing a new perspective for the study of the upper echelons theory.

Second, existing research focuses on digital transformation’s strategic effectiveness and economic consequences, with limited exploration of the antecedent factors (Cortellazzo et al., 2019; Gupta and Bose, 2022; Oh et al., 2022). This study reveals digital transformation’s driving factors and moderating conditions from decision-making and ownership perspectives. It breaks through the theoretical understanding that executives inhibit digital transformation based on risk avoidance and path dependence and proposes that celebrity CEO effects can counteract management’s psychological resistance. It expands research on digital transformation from an outcome-oriented to a causal level, providing references for future research on digital transformation strategies and empirical studies on the causes and phenomena of digital transformation.

Finally, this study uncovers celebrity CEOs’ real purpose and underlying motivations in driving companies’ digital transformation from a performance perspective. It examines the performance pressure of CEOs’ celebrity effects and the strategic decision-making tendencies under such pressures. It verifies whether there is a “face-saving” issue when implementing digital transformation strategies by celebrity CEOs (Bao et al., 2023). The study concludes that enhancing executives’ social influence is beneficial for companies to accelerate transformation, upgrading, and performance growth. This contributes to a more comprehensive and in-depth understanding of the interactions between entrepreneurs and society in related research.

### 5.2 Practical implications

The practical implications of this study are as follows. First, by revealing the impact mechanism of celebrity CEOs on digital transformation and firm performance, this study provides a theoretical basis for boards of directors to select celebrity executives based on the development needs of their firms (Chen et al., 2021). Firms can leverage celebrity CEOs’ management capabilities and advantageous resources to drive digital transformation strategies and enhance performance. However, it is important to strengthen supervision and checks on celebrity CEOs to prevent them from adopting overly risky behaviors to achieve personal goals, potentially causing losses to the firm. Second, as the proportion of ownership by controlling shareholders and institutional investors increases while they play a supervisory role, they may also excessively interfere with and restrict the discretionary power of management, thus inhibiting the promotional effect of the executive team’s rich management experience and professional skills on firm development (Lizares, 2022). This can adversely affect strategic decision-making and performance. Therefore, the problem of excessive concentration of equity ownership among listed firms in China needs to be further addressed. Finally, the digital transformation of firms is the foundation and key to developing the digital economy as a whole. However, the current digital transformation process in Chinese firms faces a bottleneck. Only 16% of the firms benefit from digital transformation, whereas many traditional firms are still in the exploratory stage because of weak digital technology foundations. Considering the risk characteristics of digital transformation as a typical strategic initiative, many firms hesitate to embrace it (Sousa and Rocha, 2019; Warner and Wäger, 2019). The positive interaction between CEOs and society as well as the enhancement of their social status, accelerates firms’ digital transformation processes (Cortellazzo et al., 2019; Katsamakas, 2022). Therefore, relevant government departments and society as a whole should fully grasp this causal relationship, bestow appropriate social identities on entrepreneurs, align corporate strategies with national policy guidelines, promote the rapid growth of the digital economy, and provide impetus for the construction of a new development pattern and the comprehensive promotion of high-quality and sustainable economic development.

### 5.3 Limitations and future research

This study had several limitations. First, we only measured the willingness and degree of digital transformation in firms using text analysis, which may not fully reflect the digital transformation process and limit the study of the relationship between celebrity CEOs, digital transformation, and firm performance. Therefore, future research should consider using more representative and easily quantifiable indicators to measure digital transformation. Second, our study does not demonstrate the actual benefits of celebrity CEOs promoting digital transformation in enhancing firm performance at different levels. Future research should investigate the extent to which celebrity CEOs enhance firm performance under varying degrees of digital transformation. Third, it did not consider the temporal sensitivity of celebrity CEO status or how celebrity status acquired at different times may affect strategic decision-making tendencies. Future research could further explore the impact of the time sensitivity of celebrity CEO status on the degree of digital transformation they promote.

## Data availability statement

The raw data supporting the conclusions of this article will be made available by the authors, without undue reservation.

## Author contributions

RW: Conceptualization, Data curation, Formal analysis, Methodology, Writing – original draft, Writing – review & editing. DS: Conceptualization, Data curation, Funding acquisition, Validation, Writing – review & editing. XH: Formal analysis, Methodology, Writing – review & editing. YL: Data curation, Validation, Writing – review & editing.
